# Cardiac β_2_-Adrenergic Receptor Phosphorylation at Ser^355/356^ Regulates Receptor Internalization and Functional Resensitization

**DOI:** 10.1371/journal.pone.0161373

**Published:** 2016-08-19

**Authors:** Xiaofang Fan, Xuejiang Gu, Ru Zhao, Qingqing Zheng, Lan Li, Wenbing Yang, Lu Ding, Feng Xue, Junming Fan, Yongsheng Gong, Yongyu Wang

**Affiliations:** 1 Institute of Hypoxia Medicine, Wenzhou Medical University, Wenzhou, Zhejiang, P.R. China; 2 Department of Endocrine and Metabolic Diseases, 1^st^ Affiliated Hospital of Wenzhou Medical University, Wenzhou, Zhejiang, P.R. China; 3 Department of Pathology, Shantou University Medical College, Shantou, Guangdong, P.R. China; University of Houston, UNITED STATES

## Abstract

Previous studies have demonstrated that β_2_-adrenergic receptors (β_2_ARs) can be phosphorylated by G protein-coupled receptor kinases (GRKs) and protein kinase A (PKA), affecting β_2_AR internalization and desensitization. However, the exact physiological function of β_2_ARs in cardiomyocytes is unknown. In this study, we showed that neonatal mouse cardiomyocytes had different contraction and internalization responses to sustained or repeated, transient agonist stimulation. Specifically, short-time stimulation (10 min) with epinephrine or norepinephrine increased the cardiomyocyte contraction rate, reaching a maximum at 5 min, followed by a slow decline. When the agonist was re-added after a 60-min wash-out period, the increase in the cardiomyocyte contraction rate was similar to the initial response. In contrast, when cardiomyocytes were exposed continuously to epinephrine or norepinephrine for 60 min, the second agonist stimulation did not increase the contraction response. These results indicated that continuous β_2_AR stimulation caused functional desensitization. Phosphorylation of β_2_ARs at serine (Ser)^355/356^ GRK phosphorylation sites, but not at Ser^345/346^ PKA phosphorylation sites increased with continuous epinephrine stimulation for 60 min. Accordingly, β_2_AR internalization increased. Interestingly, β_2_AR internalization was blocked by mutations at the GRK phosphorylation sites, but not by mutations at the PKA phosphorylation sites. Furthermore, inhibition of β_2_AR dephosphorylation by okadaic acid, a phosphatase 2A inhibitor, impaired the recovery of internalized β_2_ARs and reduced the cardiomyocyte contraction rate in response to epinephrine. Finally, epinephrine treatment induced the physical interaction of β-arrestin with internalized β_2_ARs in cardiomyocytes. Together, these data revealed the essential role of the Ser^355/356^ phosphorylation status of β_2_ARs in regulating receptor internalization and physiological resensitization in neonatal cardiomyocytes to contraction functions.

## Introduction

β_2_-Adrenergic receptors (β_2_ARs) belong to the G protein-coupled receptor (GPCR) superfamily and play significant roles in regulating cardiovascular and airway functions. Dysregulation of β_2_ARs is associated with heart failure, asthma, and other diseases [1–3]. Activation of β_2_ARs induces receptor binding to G proteins followed by dissociation of the G_α_ and G_βγ_ subunits. β_2_ARs couple to both Gs and Gi proteins that regulate adenylyl cyclase generating cAMP to activate protein kinase A (PKA), which phosphorylates downstream proteins, thus mediating various physiological functions [4].

Activation of β_2_ARs leads to rapid receptor phosphorylation by PKA and G protein-coupled receptor kinases (GRKs), which leads to receptor binding to β-arrestin, terminates G protein-mediated signaling, and facilitates receptor endocytosis resulting in receptor desensitization and internalization [5]. This is one of the reasons why prolonged or repeated use of β_2_ agonists leads to a loss of their effect. Once β_2_ARs are dephosphorylated through protein phosphatase 2A (PP2A), the receptor becomes resensitized to agonist stimulation. β_2_ARs can be phosphorylated on their C termini and intracellular loops, such as Ser^261/262/345/346^ by PKA and Ser^355/356/360/364^ by GRKs, and the distinct phosphorylation sites mediate different intracellular signaling pathways and physiological functions [6].

Much of the work on β_2_AR phosphorylation, internalization, and resensitization has been performed in transiently transfected cells. There are few data from primary cells or animal models, which limits our understanding of the physiology of β_2_AR phosphorylation. At the same time, although the mechanism of β_2_AR desensitization is well characterized, less is known about β_2_AR resensitization, particularly the role of β_2_AR dephosphorylation in functional resensitization and internalization in primary cardiomyocytes. Recent studies have revealed that resensitization should be considered equally important as desensitization for the regulation of β_2_AR functions [7].

Phosphorylation of Ser^261/262^ in cardiomyocytes has been previously demonstrated, and was shown to be involved in regulating the contraction rate response and Gi signaling coupling [7]. At the same time, we could not confirm phosphorylation of Ser^360/364^ in cardiomyocytes owing to the lack of phosphor-specific antibodies for these sites. Therefore, in the current study, we focused on Ser^345/346^ and Ser^355/356^. We found that the Ser^355/356^ phosphorylation sites on β_2_ARs have a crucial role in regulating β_2_AR internalization and resensitization in neonatal mouse cardiomyocytes.

## Materials and procedures

### Isolation and culture of neonatal cardiac myocytes

All animal study protocols were approved by the Institutional Animal Care and Use Committee of the Wenzhou Medical University and complied with the regulations of the Ministry of Health, China, and the USA National Institutes of Health Guidelines for the use and care of laboratory animals. All efforts were made to minimize animal suffering and to reduce the number of animals used.

β1/β2AR double knockout (DKO) mice (Adrb1tm1BkkAdrb2tm1Bkk/J) were purchased from the Jackson Laboratory (Bar Harbor, ME, USA) [8]. β1AR knockout chimeric mice were crossed originally with C57BL/6J × DBA/2 F1hybrid mice [9]. Neonatal mice (less than 12 h old) were anesthetized with isoflurane and decapitated, and the hearts were excised quickly. Cardiac myocytes were isolated and cultured as described previously [10]. Briefly, each isolated heart was cut into pieces and incubated two times with papain (Sigma-Aldrich, St. Louis, MO, USA) with shaking at 37°C for 5 min. The tissue was then pipetted vigorously to disperse it into single cells. After removing the digestion solution, the cells were resuspended in Dulbecco’s modified Eagle’s medium and plated onto a gelatin-coated dish after filtering through a cell strainer. After 1 h, the myocytes were collected and placed onto new dishes.

### Site-directed mutagenesis and recombinant adenoviruses

To determine the role of β_2_AR phosphorylation at the GRK phosphorylation sites Ser^355^ and Ser^356^, a pcDNA 3.1-FLAG-tagged murine β_2_AR was used as a template for mutagenesis with the QuickChange site-directed mutagenesis kit (Stratagene, La Jolla, CA, USA). Both serines were replaced with alanines according to the manufacturer’s instructions. In addition, the PKA phosphorylation sites (Ser^345^ and Ser^346^) on β_2_AR were mutated by replacing the two serines with alanines. The mutant plasmids were named GRK2A and PKA2A, respectively. Mutant β_2_AR constructs were confirmed by sequencing and transformed into adenovectors to produce adenoviruses using the AdEasy Adenoviral Vector System (Agilent Technologies, Santa Clara, CA). Virus titers were assessed by determining the receptor expression levels by both western blot and ligand binding assays, as described previously [11].

### Neonatal cardiac myocyte contraction assay

Measurements of the spontaneous contraction of cardiac myocytes were performed as described previously with modification [11]. Briefly, after isolation of the cardiomyocytes from neonatal heart tissues and a 1-h pre-culture in dishes, the cells were collected by centrifugation. The cells from each heart sample were resuspended in 30 μL of medium, and 10 μL of the concentrated cell suspension (approximately 3 × 10^5^ cells) was placed in the center of 35-mm dishes and cultured for 24 h. Then, the medium was changed and the cells were cultured for another 24 h to obtain a uniformly beating syncytium. The cells were equilibrated in a chamber on the stage of an inverted microscope at 37°C for 10 min before monitoring of the contraction rate. For the desensitization/resensitization assay, the contraction rates of the syncytia were recorded every 2 min for 10 min after the addition of epinephrine (Epi, 10 μM) or norepinephrine (NE, 10 μM) (Sigma-Aldrich). Then, the medium was changed or the cells were kept in the medium containing stimulator for 60 min, after which the contraction rates were recorded for another 10 min following a second stimulation. Data were recorded using the software on the computer. For measurements of the effect of repeated stimulation with agonist Epi or NE, the contraction response of mouse neonatal cardiomyocytes was recorded in real-time for 40 min.

### β_2_AR internalization and recycling assay

Neonatal cardiac myocytes isolated from DKO mice were transfected with plasmids encoding wild-type or phosphorylation-site mutant β_2_ARs at a multiplicity of infection (MOI) of 100 for 24 h. After serum starvation for 2 h, the myocytes were stimulated with 10 μM Epi for different periods. For receptor recycling, the cells were stimulated with Epi for 10 min, rinsed, and refed with serum-free medium for different periods after removal of Epi for 1 h. The cells were then fixed, permeabilized, incubated with anti-FLAG antibody, and visualized using an Alexa 488-conjugated goat anti-mouse antibody (Invitrogen, Carlsbad, CA, USA). Fluorescence images were taken with a camera on a Zeiss Axioplan 2 microscope and analyzed with Metamorph software (Molecular Devices, Sunnyvale, CA, USA). To quantify the surface receptor level, a fluorescence-linked immunosorbent assay (FLISA) was applied as described previously [12]. Briefly, neonatal cardiomyocytes cultured in poly-lysine-coated 12-well plates were transfected with FLAG-β_2_AR adenovirus at a MOI of 100 for 24 h, stimulated with Epi under the indicated conditions, and fixed with 4% paraformaldehyde in phosphate-buffered saline (PBS). Without permeabilization, the cells were blocked directly with 2.5% goat serum in PBS, and then stained with Alexa 488-conjugated M1 antibody (Sigma) at a concentration of 1 μg/mL for 30 min at room temperature. The unbound antibody was removed by washing four times with PBS. The cells were harvested with 1% SDS in PBS, and the intensity of Alexa 488 emission (495 to 580 nm) was measured using a spectrofluorometer with an excitation wavelength of 485 nm and an integration time of 0.3 s/nm. The fluorescence intensity was normalized by subtracting the background from cells without M1 antibody.

### Beta_2_AR phosphorylation assay by western blot analysis

Neonatal cardiac myocytes were transfected with FLAG-tagged wild-type or mutant β_2_ARs at a MOI of 100 for 48 h. Then, the cells were serum-starved for 2 h and stimulated with 10 μM Epi for the indicated times. The cells were lysed in radioimmunoprecipitation assay lysis buffer (Thermo Scientific, Rockford, IL, USA) at 4°C. Samples were clarified by centrifugation at 16,100 × *g* for 10 min, and the protein concentration of the supernatant was measured by the bicinchoninic acid assay (Thermo Scientific) according to the manufacturer's instructions. Samples were resolved by SDS-PAGE for western blotting. Phosphorylation of β_2_AR by GRK at Ser^355/356^ or by PKA at Ser^345/346^ was detected with phospho-specific AR antibodies (Santa Cruz Biotechnology, Santa Cruz, CA, USA). IRDye 800CW-conjugated donkey anti-rabbit antibody (LI-COR, Lincoln, NE, USA) was used as a secondary antibody. Western blots were visualized with an Odyssey CLx infrared imager (LI-COR) and quantified after normalization against baseline levels.

### Immunofluorescence and confocal microscopy

Cardiac myocytes were cultured on coverslips in 6-well plates and transfected with wild-type or mutant β_2_AR recombinant adenoviruses at a MOI of 100 for 24 h. The cells were serum-starved for 2 h before stimulation with 10 μM Epi for the indicated times. Then, the cells were fixed with 4% paraformaldehyde for 20 min at room temperature, permeabilized, and incubated with an anti-FLAG antibody overnight at 4°C. After washing with PBS, the cells were incubated with an Alexa 488-conjugated goat anti-mouse secondary antibody for 1 h at room temperature. β_2_AR recycling was assessed by washing out the agonists after 10 min and allowing receptor recovery for an additional 60 min. Images were obtained using a Zeiss Axioplan 2 microscope with Metamorph software, or a Zeiss 710 confocal microscope.

### Statistical analysis

Curve-fitting and statistical analyses were performed using Prism (GraphPad Software, San Diego, CA, USA). All experiments were performed independently at least three times. Data are presented as the mean ± standard deviation (SD). Statistical significance was determined using ANOVA or Student’s *t*-test; a value of *P* < 0.05 was considered significant.

## Results

### Neonatal cardiac myocyte contraction response to Epi or NE under different stimulation conditions

**Fig 1:**
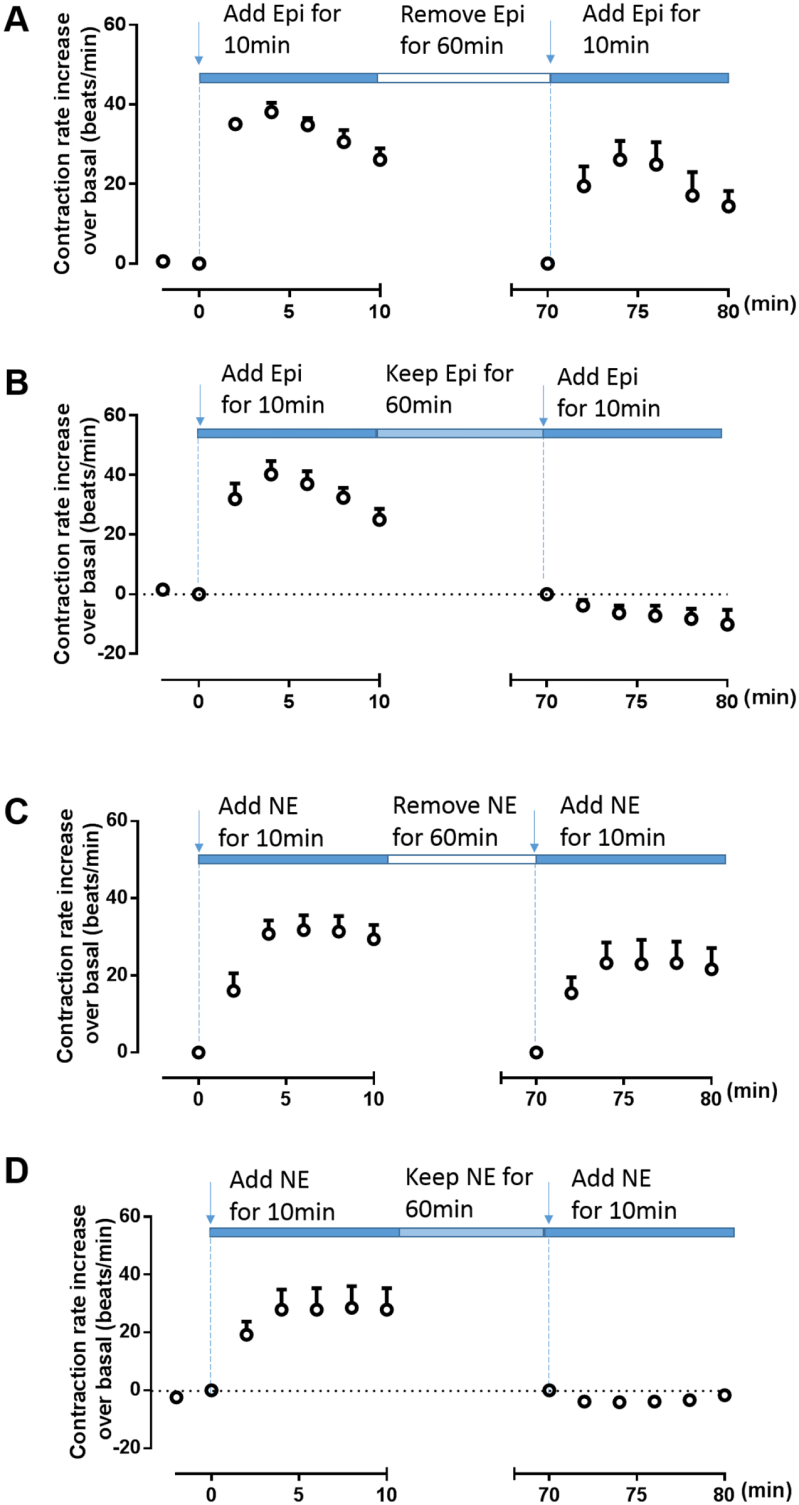
Cardiomyocyte contraction response to epinephrine or norepinephrine under different stimulation conditions. Cardiac myocytes isolated from β1AR knockout mice that express endogenous β_2_AR were stimulated with 10 μM epinephrine (Epi) or norepinephrine (NE) for 10 min and then re-challenged with (B, D) or without (A, C) Epi or NE for 60 min. The Epi- (A, B) or NE- (F, H) induced contraction rates were recorded. Data are shown as means ± SDs from three independent experiments.

Short-time stimulation (10 min) with Epi or NE rapidly increased the neonatal cardiomyocyte contraction rate, reaching a maximum at 5 min, followed by a slow decline. When the agonist was removed for 60 min and then re-added at the same concentration, the increase in the cardiomyocyte contraction rate was similar to the initial response (Fig 1A and 1C). However, when the cells were exposed continuously to Epi or NE for 60 min beyond the initial 10-min stimulation, a second agonist stimulation did not increase the contraction response and, in fact, weakened the spontaneous contractions to below the basal level (Fig 1B and 1D). These results suggested that continuous β_2_AR stimulation caused functional desensitization of cardiac myocytes, which prevented contraction rate increases in response to a second agonist challenge. In contrast, removal of the β_2_AR agonist after 10 min allowed recovery of the contraction response, indicating functional resensitization of the receptor. This suggested that these treatment scenarios provided excellent *in vitro* cell models for the β_2_AR desensitization/resensitization response in cardiac myocytes.

### Epi-induced internalization and recycling of neonatal cardiomyocyte β_2_AR under different stimulation conditions

**Fig 2:**
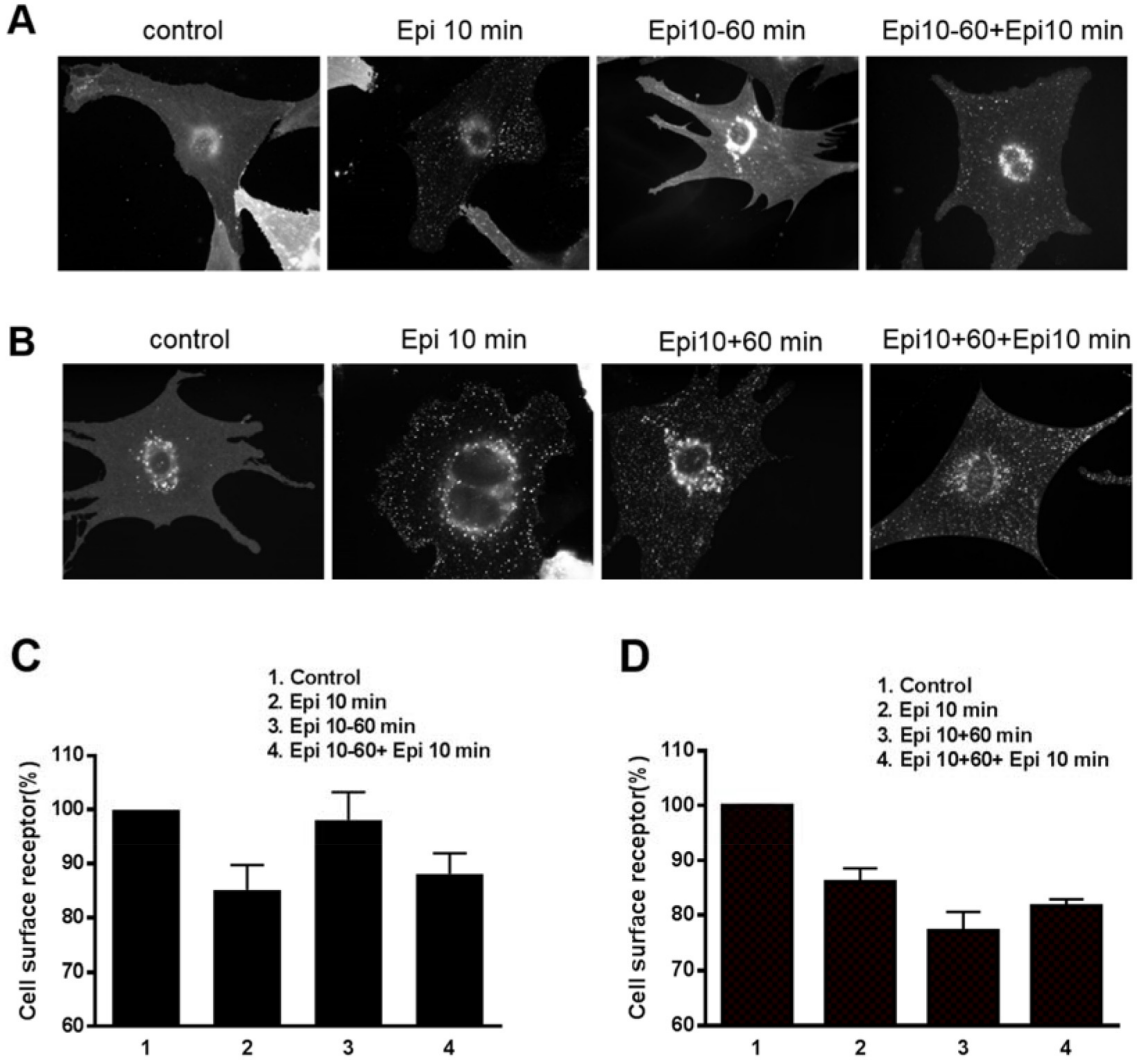
Epinephrine-induced internalization and recycling of β_2_ARs under different stimulation conditions in cardiomyocytes. Neonatal cardiomyocytes isolated from β_1_β_2_AR double-knockout mice were transfected with FLAG-tagged wild-type β_2_AR at a multiplicity of infection of 100 for 24 h. After serum starvation for 2 h, the cardiac myocytes were stimulated with 10 μM epinephrine (Epi). Epi was then removed (A) or retained (B) for 60 min followed by Epi re-stimulation for another 10 min. Cardiomyocytes were fixed and stained with anti-FLAG and Alexa 488-conjugated goat anti-mouse antibodies. (A) Punctate intracellular staining of FLAG-tagged β_2_ARs was observed after 10 min of Epi stimulation. The Epi-activated FLAG-β_2_AR efficiently recycled back to the cell surface after removal of the drug. Epi-activated FLAG-β_2_AR was rapidly internalized after secondary stimulation. (B) When Epi was present continually, the receptor was not recycled but remained internalized. Photographs representative of 3 different preparations of cardiomyocytes are shown. The cell surface receptors were quantified by a fluorescence-linked immunosorbent assay (FLISA) with Epi treatment for 10 min and then Epi was removed (C) or retained (D) for 60 min followed by Epi re-stimulation for another 10 min.

The desensitization/resensitization of β_2_ARs is related to receptor internalization and recycling. We assessed these processes in our *in vitro* cardiac myocyte model using the same contraction response conditions as for Epi stimulation. DKO cardiac myocytes transfected with FLAG-β_2_AR adenovirus were treated with Epi for 10 min. Internalization of FLAG-β_2_ARs was observed clearly as punctate intracellular staining (Fig 2). Interestingly, the intracellular punctate staining disappeared after Epi withdrawal for 60 min, suggesting that the receptors were recycled back to the cell surface. When the myocytes were stimulated with Epi a second time, the β_2_ARs were again rapidly (10 min) internalized (Fig 2A and 2C). In contrast, in cells exposed to Epi for 60 min, the internalized FLAG-β_2_ARs aggregated within the cytoplasm rather than recycling back to the cell surface. When these cells were stimulated with Epi a second time, the receptors remained in the cytoplasm (Fig 2B and 2D). This finding confirmed that hyper-stimulation by a β_2_AR agonist led to receptor desensitization.

### Mutation at the β_2_AR Ser^355/356^ sites impaired internalization of β_2_ARs in neonatal cardiac myocytes

**Fig 3:**
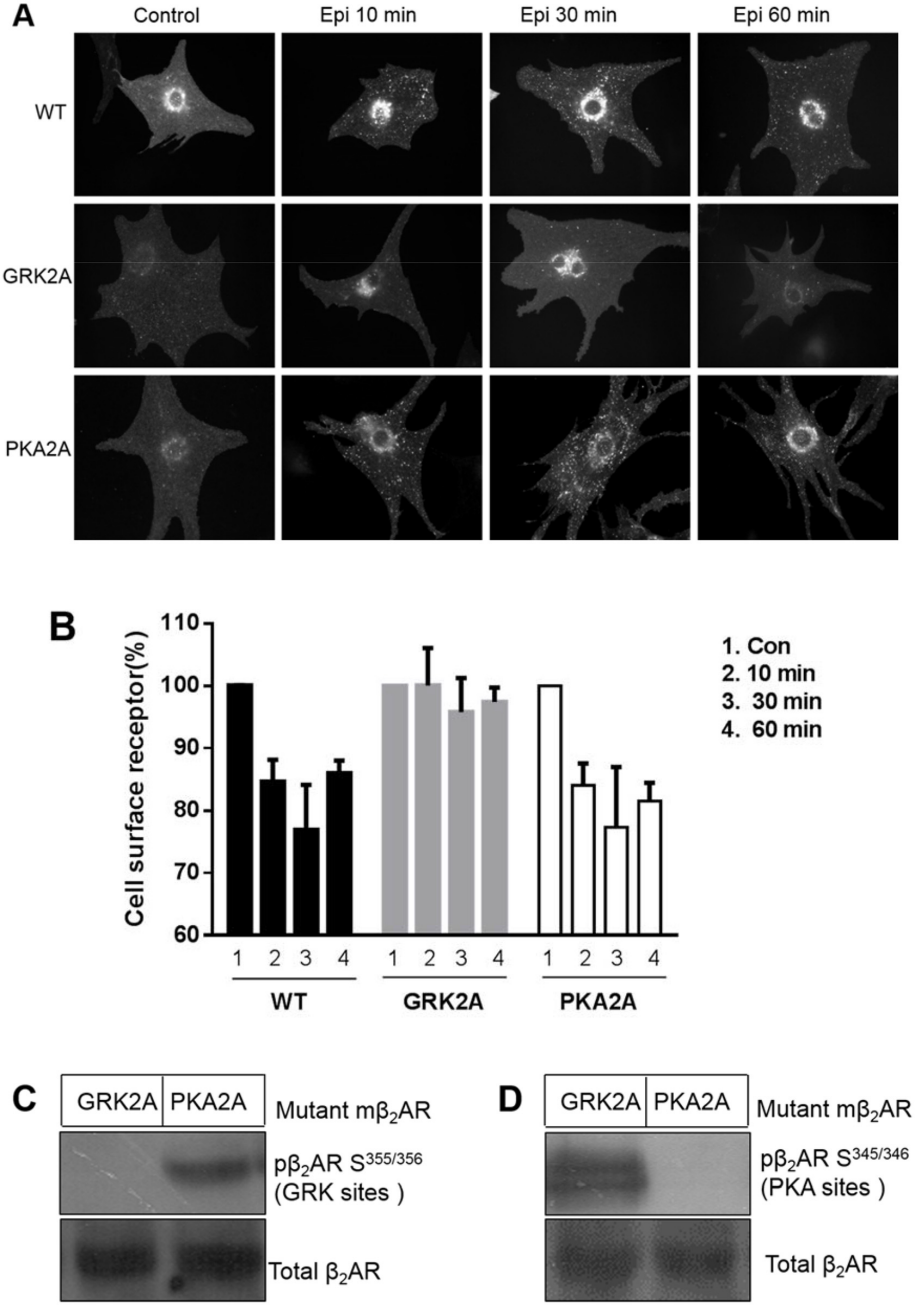
Mutation of β_2_AR S^355/356^ sites impaired internalization of β_2_ARs. Wild-type and two mutant forms of β_2_AR (GRK2A and PKA2A) were expressed in neonatal cardiomyocytes isolated from β_1_β_2_AR double-knockout mice by adenovirus transfection for 2 days. (A) The cells were stimulated with Epi (10 μM) for 10, 30, or 60 min. Compared to wild-type, GRK2A β_2_AR (mutation at S^355/356^), but not PKA2A β_2_AR (mutation at S^345/346^), impaired receptor internalization. Photographs representative of 3 different preparations of cardiomyocytes are shown. (B) The cell surface receptors of wild-type or mutant β_2_AR were quantified by FLISA upon Epi stimulation for the indicated times. The quantitative data represent the means ± SDs of at least 3 different experiments. (C) Cardiomyocytes from β_1_β_2_AR double-knockout mice were transfected with GRK2A and PKA2A mutant β_2_ARs for two days and then lysed with radioimmunoprecipitation assay buffer. The lysates were subjected to SDS-PAGE followed by immunoblot analysis using a polyclonal anti-phosphoserine (355, 356)-specific or anti-phosphoserine (345, 346)-specific β_2_AR antibody. Phospho-β_2_AR Ser^355/356^ (GRK sites) was not observed in GRK2A (mutation of β-AR at Ser^355/356^), but was present in PKA2A (mutation of β-AR at Ser^345/346^). (D) In contrast, Phospho-β_2_AR Ser^345/346^ was not observed in PKA2A (mutation of β-AR at Ser^345/346^), but was present in GRK2A (mutation of β-AR at Ser^355/356^).

To further assess the importance of β_2_AR phosphorylation for receptor internalization, we mutated Ser^355/356^ (GRK2A) or Ser^345/346^ (PKA2A) to alanines to generate β_2_ARs with defective phosphorylation sites. When these mutant β_2_ARs were expressed in DKO cardiac myocytes and stimulated with Epi, the PKA2A mutant receptors generated punctate intracellular staining similar to that in wild-type cells. In contrast, the GRK2A mutant receptors showed impaired internalization (Fig 3, middle panel). In addition, we confirmed that mutation at the Ser^355/356^ sites of β_2_AR completely blocked the Ser^355/356^ phosphorylation after exposure to Epi, while the PKA2A mutant showed no phosphorylation effects (Fig 3B). Thus, PKA2A but not GRK2A mutations impair β_2_AR phosphorylation upon Epi treatment (Fig 3C). These results confirmed that the phosphorylation status of Ser^355/356^ was essential for β_2_AR internalization.

### Epi-induced β_2_AR phosphorylation at Ser^355/356^ but not at Ser^345/346^ was related to receptor internalization in neonatal cardiac myocytes

**Fig 4:**
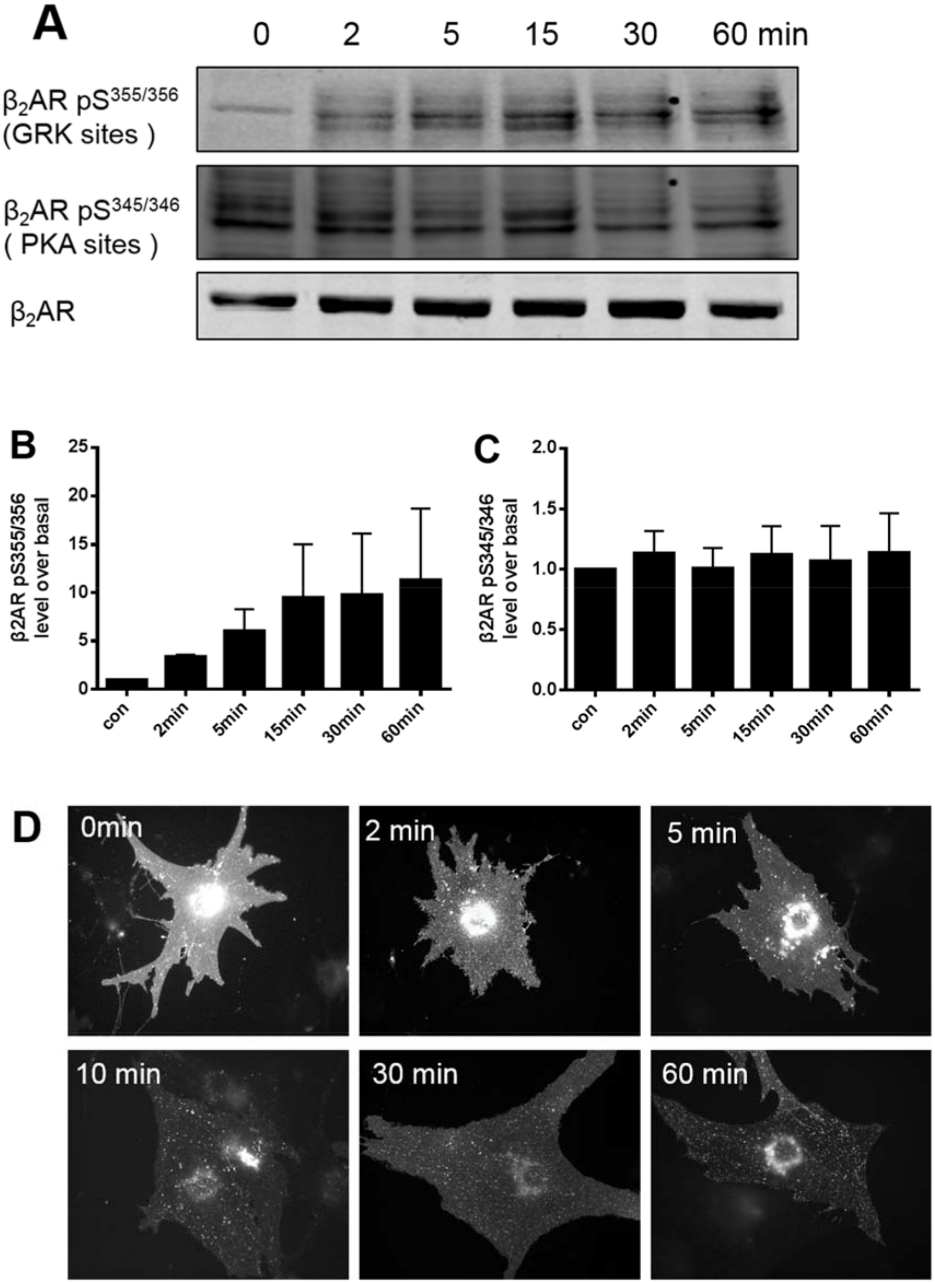
Epinephrine-induced β_2_AR phosphorylation at S^355/356^ (GRK) but not at S^345/346^ (PKA) is related to receptor internalization in cardiac myocytes. (A) Neonatal cardiomyocytes from β_1_β_2_AR double-knockout mice were transfected with the β_2_AR adenovirus for 2 days and then stimulated with epinephrine (Epi; 10 μM) for 2, 5, 15, 30, or 60 min. Cell lysates were subjected to SDS-PAGE followed by immunoblot analysis using polyclonal anti-phosphoserine-(355, 356) or -(345, 346)-specific β_2_AR antibodies. The blots were stripped and probed with an anti-C-terminal β_2_AR antibody to visualize total β_2_ARs. (B) and (C) Relative quantitative analysis of the levels of phosphor-β_2_AR was performed by normalizing the phosphor-β_2_AR signal against the total β_2_AR. (D) Punctate intracellular staining of FLAG-β_2_ARs was observed at all time points of Epi stimulation, indicating that Epi induced receptor internalization.

To further assess whether phosphorylation of β_2_ARs correlated with desensitization and resensitization, cardiac myocytes from neonatal DKO mice were transfected with β_2_ARs and stimulated with Epi. There was increased β_2_AR phosphorylation at Ser^355/356^ but not at Ser^345/346^ following 2 to 60 min of continuous stimulation with Epi (Fig 4A–4C). Correspondingly, the internalized β_2_ARs accumulated time-dependently within the cytoplasm during long, uninterrupted stimulation (Fig 4D). This suggested that Epi promotes GRK phosphorylation at Ser^355/356^, which was vital for receptor internalization in cardiomyocytes.

### Blocking phosphorylation of β_2_ARs at Ser^355/356^ sites impaired their internalization and the contraction response

**Fig 5:**
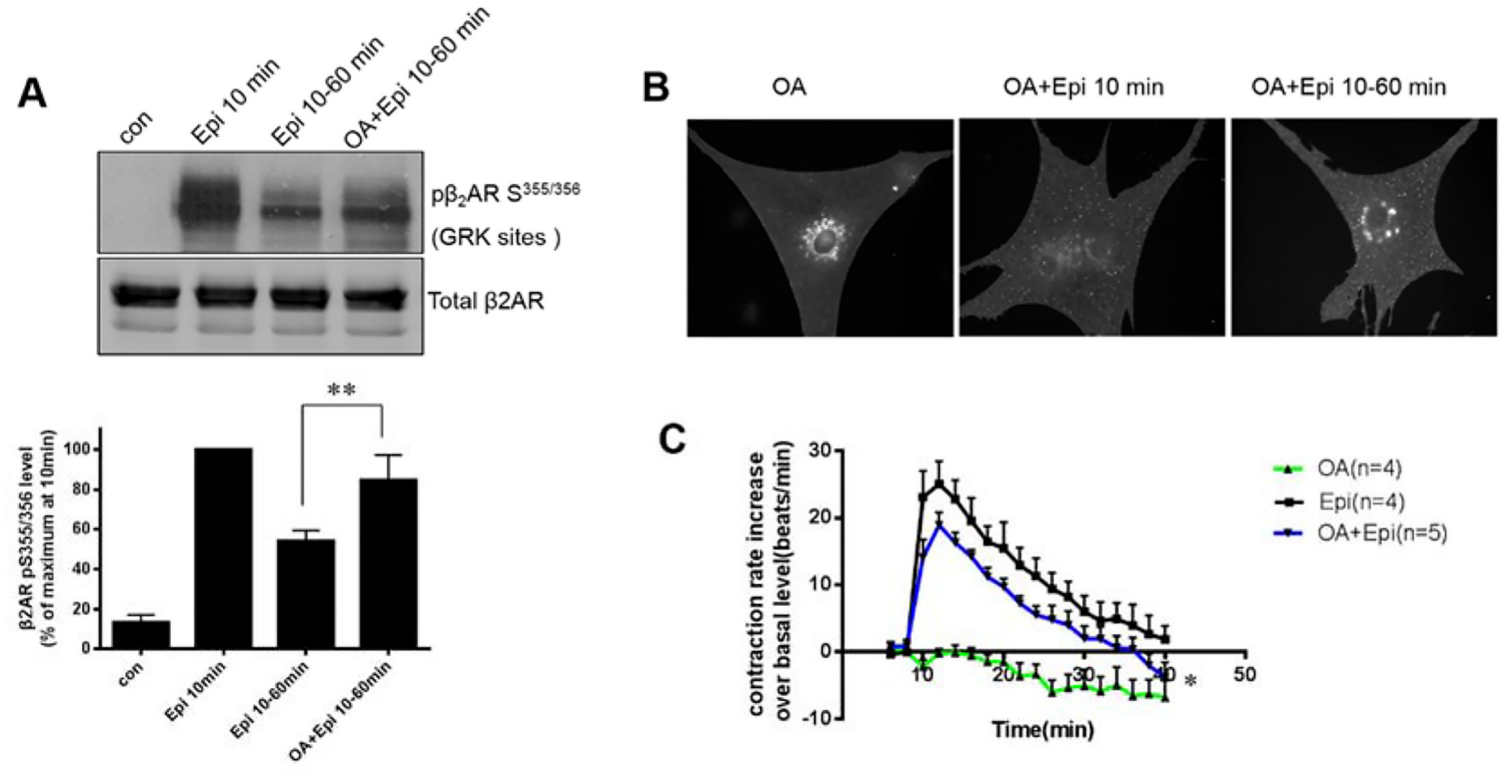
Blocking of phosphorylation of the β_2_AR S^355/356^ sites inhibits the contraction response and impairs internalization of β_2_AR. Cardiomyocytes from β_1_β_2_AR double-knockout mice were transfected with the β_2_AR adenovirus for 2 days and then stimulated with epinephrine (Epi) (10 μM) for 10 min with or without okadaic acid (OA) pretreatment. Epi was then removed for 60 min. (A) Phosphorylation of the β_2_ARs at Ser^355,356^ and (B) internalization of the β_2_ARs were examined. (C) Cardiac myocytes isolated from β_1_AR knockout mice were used for the contraction rate assay. Cells were stimulated with Epi with or without OA pre-treatment. The contraction response curves represent the means ± SDs of *n* beating dishes from at least 3 different neonatal cardiomyocyte preparations. *p < 0.05 vs. Epi-treated group (two-way ANOVA). **p < 0.05 vs. group without OA treatment (Student’s *t*-test).

To confirm whether phosphorylation of β_2_ARs at Ser^355/356^ was critical to receptor desensitization and resensitization, cardiomyocytes overexpressing β_2_ARs were pretreated with okadaic acid (OA), a PP2A inhibitor, prior to Epi stimulation. Epi induced rapid (10 min) receptor phosphorylation at Ser^355/356^ (Fig 5A). After removal of the drug, β_2_ARs underwent dephosphorylation over 60 min. Pretreatment with OA attenuated β_2_AR dephosphorylation (Fig 5A). In addition, the blockade of β_2_AR dephosphorylation by OA resulted in the accumulation of phosphorylated β_2_ARs in the cytoplasm after withdrawal of Epi for 60 min (Fig 5B). Interestingly, OA treatment not only reduced receptor recycling to the cell surface but also diminished the contraction response to Epi treatment (Fig 5C).

### Epinephrine induced an interaction between β_2_ARs and β-arrestin 2

**Fig 6:**
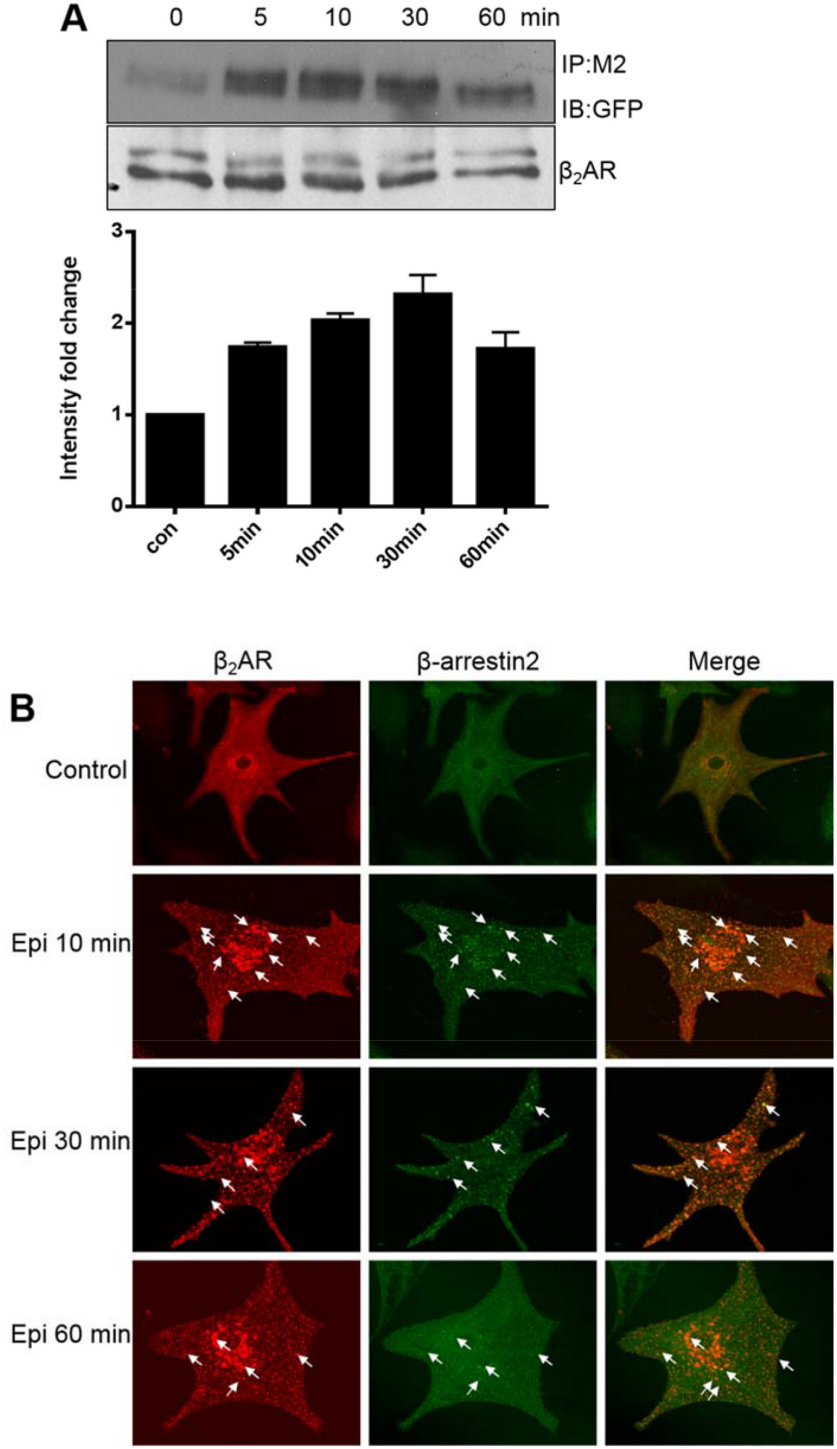
Epinephrine induces interactions between β_2_ARs and β-arrestin 2. Cardiac myocytes from β_1_β_2_AR double-knockout mice were transfected to express FLAP-β_2_AR and β-arrestin-GFP for 2 days, and then stimulated with epinephrine (Epi) for 5, 10, 30, or 60 min. (A) Cell lysates were immunoprecipitated with an anti-Flag-m2 antibody. Immunoblot analyses were performed using anti-GFP or β-arrestin antibodies. (B) Cells were fixed for immunofluorescence staining. Intracellular localization of Flag-mβ_2_AR and β-arrestin-GFP was observed at the indicated times of Epi stimulation. The results presented are representative of at least 3 repeated experiments. Arrows show co-localization of internalized β_2_ARs and β-arrestin.

To determine the interaction between β_2_ARs and the cytoplasmic protein β-arrestin 2 after internalization, we constructed two specifically labeled plasmids that expressed β_2_ARs and β-arrestin 2. Two days after transfection, DKO cardiac myocytes that highly expressed FLAG-mβ_2_AR and β-arrestin-GFP were stimulated with Epi for 5 to 60 min. Co-immunoprecipitation demonstrated that phosphorylated β_2_ARs underwent internalization and interacted with β-arrestin 2 (Fig 6A). Furthermore, immunofluorescence experiments verified that β_2_ARs interacted and co-located with β-arrestin 2, and that the interacted proteins time-dependently assembled in the cytoplasm (Fig 6B). This suggested that β-arrestin 2 played a significant role in the process of receptor dephosphorylation.

## Discussion

We report the functional resensitization of β_2_ARs as assessed by a neonatal cardiomyocyte contraction assay *in vitro*, as well as the receptor internalization response under repeated agonist stimulation. These responses involved β_2_AR phosphorylation on the Ser^355/356^ sites. Desensitization of GPCRs is an important physiological feedback mechanism that protects cells against acute and chronic receptor overstimulation [11]. Indeed, we found that the continuous presence of Epi for 60 min caused β_2_AR functional desensitization by showing that a second β_2_AR stimulation did not increase the cardiomyocyte contraction rate after continuous stimulation (Fig 1B). In contrast, early withdrawal of Epi (10 min) caused functional resensitization of β_2_AR as shown by a similar contraction response when compared to the initial stimulation with Epi (Fig 1A).

Sustained stimulation of β_2_ARs with Epi promoted receptor internalization and their retention in the cytoplasm (Fig 2B), while withdrawal of Epi helped the receptors recycle back to the cell surface and re-internalize with a second stimulation (Fig 2A). Importantly, we found that phosphorylation of β_2_ARs at Ser^355/356^ sites was critical to this physiological resensitization and internalization. Specifically, elimination of β_2_AR phosphorylation at Ser^355/356^ but not Ser^345/346^ by mutation to alanine completely blocked receptor internalization, even with sustained Epi stimulation (Fig 3A). At the same time, inhibiting dephosphorylation of β_2_AR at Ser^355/356^ by OA treatment decreased the contraction response in cardiomyocytes (Fig 5C).

In HEK293 cells, Ser^355/356^ plays a pivotal role in desensitization and internalization of β_2_ARs [13]. In the present study, we confirmed that phosphorylation of β_2_ARs at Ser^355/356^ contributed to receptor internalization in primary neonatal cardiomyocytes. This finding is consistent with previous data from cardiomyocytes [11]. GRK2 and GRK5 are major GRKs expressed in the heart, while GRK6 is usually expressed at low levels in this tissue [12]. We, and others, have reported that GRK2 could phosphorylate β_2_AR at Ser^355/356^ affecting β_2_AR trafficking and the contraction rate response in cardiomyocytes [14]. It has been suggested that GRK6 promotes phosphorylation of β_2_AR at Ser^355/356^, while GRK2 inhibits phosphorylation at these sites in HEK293 cells stimulated with isoproterenol [6]. Furthermore, these GRK6 sites were primarily responsible for β_2_AR desensitization and β-arrestin-mediated ERK activation, but not for internalization.

Previous observations suggested that β_2_ARs could be phosphorylated by different GRKs in various cells or tissues, thereby mediating distinct physiological functions [15,16]. When we eliminated the Ser^345/346^ PKA phosphorylation sites, the internalization of β_2_ARs did not change, indicating that these phosphorylation sites are not necessary for receptor internalization in cardiomyocytes. With Epi continually present in the medium for 60 min, the phosphorylation state of β_2_AR at Ser^355/356^ in cardiomyocytes was sustained, and the receptor was retained in the cytoplasm rather than recycled (Fig 4D). In this condition, Ser^345/346^ sites were not phosphorylated, confirming that phosphorylation of β_2_ARs at these sites might not be involved in receptor internalization.

Although 13 β_2_AR phosphorylation sites have been identified in HEK293 cells using isoproterenol treatment [6], the phosphorylation pattern of β_2_ARs in cardiomyocytes following Epi stimulation was unknown. In addition, whether all 13 phosphorylation sites were essential for receptor internalization and resensitization remained to be elucidated. In the present work, we found that dephosphorylation at Ser^355/356^ sites 60 min after agonist removal allowed a second functional response, including receptor re-internalization and an increased contraction rate. Therefore, while the total phosphorylation pattern was unclear in cardiac β_2_ARs with Epi stimulation, the β_2_AR Ser^355/356^ phosphorylation state definitely contributed to receptor desensitization and resensitization.

Our results also indicated that elimination of sustained agonist stimulation promoted receptor dephosphorylation at Ser^355/356^ and recovery of the receptor and cell functions in response to a second agonist challenge. PP2A is important for regulating the dephosphorylation of β_2_AR in cardiomyocytes [17]. The balance between GRK and PP2A determines the phosphorylation status of β_2_AR and affects different functions including internalization and resensitization. For example, β_2_AR resensitization is defective in severe asthma [18]. In this case, PP2A activity is reduced, possibly due to decreased β_2_AR dephosphorylation at Ser^355/356^, leading to β_2_AR dysfunction. The molecular mechanisms of βAR resensitization have been reported to also involve the inhibition of phosphoinositide 3-kinase-γ in the heart [7]. This inhibition increased PP2A activity and effectively dephosphorylated βARs. β_2_AR dephosphorylation at Ser^355/356^ was involved in this process, which is consistent with our observations. In addition, some scaffolding A-kinase-anchoring proteins may regulate βAR resensitization by targeting the phosphatase-receptor complex [19,20]. However, additional details of the resensitization mechanism of βARs in cardiomyocytes need to be elucidated.

It is believed that β_2_AR internalization is required for resensitization. Once internalized, receptors undergo dephosphorylation in the early endosomes by PP2A [17] before they recycle back to the plasma membrane [3]. β-Arrestin plays a central role during this receptor internalization (sequestration) process that contributes to normal β_2_AR dephosphorylation and resensitization [21]. β-Arrestin- and clathrin-coated vesicles appear to be needed for β_2_AR internalization, which targets receptors to the endosome for dephosphorylation and resensitization [21]. We confirmed that β-arrestin physically interacted with Ser^355/356^ phosphorylated β_2_ARs in cardiomyocytes. Additionally, we found that β-arrestin was bound to β_2_ARs for a longer time in the continuous presence of the agonist, slowing the dephosphorylation process, which might block the functional resensitization of the receptors.

Together, these data revealed that the Ser^355/356^ phosphorylation status of β_2_ARs in neonatal cardiomyocytes is critical for receptor internalization and functional resensitization. This finding suggested that the physiological function of β_2_ARs not only depends on agonist stimulation, but also on specific conditions in cells or tissues, as well as the current receptor phosphorylation modification status.
